# The role of active case finding in reducing patient incurred catastrophic costs for tuberculosis in Nepal

**DOI:** 10.1186/s40249-019-0603-z

**Published:** 2019-12-03

**Authors:** Suman Chandra Gurung, Kritika Dixit, Bhola Rai, Maxine Caws, Puskar Raj Paudel, Raghu Dhital, Shraddha Acharya, Gangaram Budhathoki, Deepak Malla, Jens W. Levy, Job van Rest, Knut Lönnroth, Kerri Viney, Andrew Ramsay, Tom Wingfield, Buddha Basnyat, Anil Thapa, Bertie Squire, Duolao Wang, Gokul Mishra, Kashim Shah, Anil Shrestha, Noemia Teixeira de Siqueira-Filha

**Affiliations:** 1 Birat Nepal Medical Trust, Kathmandu, Nepal; 2 Department of Clinical Sciences, Liverpool School of Tropical Medicine, Liverpool, UK; 3 KNCV Tuberculosis Foundation, The Hague, Netherlands; 4 Department of Public Health Sciences, Karolinska Institutet, Stockholm, Sweden; 5 Research School of Population Health, Australian National University, Canberra, Australia; 6 University of St Andrews, St Andrews, UK; 7 University of Liverpool, Liverpool, UK; 8 Oxford University Clinical Research Unit, Kathmandu, Nepal; 9 National Tuberculosis Centre, Bhaktapur, Nepal; 10 Nick Simons Institute, Lalitpur, Nepal; 11 Institute for Health Technology Assessment, Porto Alegre, Brazil

**Keywords:** Tuberculosis, Case finding, Cost, Catastrophic cost, Patient-incurred cost, Nepal

## Abstract

**Background:**

The World Health Organization (WHO) End TB Strategy has established a milestone to reduce the number of tuberculosis (TB)- affected households facing catastrophic costs to zero by 2020. The role of active case finding (ACF) in reducing patient costs has not been determined globally. This study therefore aimed to compare costs incurred by TB patients diagnosed through ACF and passive case finding (PCF), and to determine the prevalence and intensity of patient-incurred catastrophic costs in Nepal.

**Methods:**

The study was conducted in two districts of Nepal: Bardiya and Pyuthan (Province No. 5) between June and August 2018. One hundred patients were included in this study in a 1:1 ratio (PCF: ACF, 25 consecutive ACF and 25 consecutive PCF patients in each district). The WHO TB patient costing tool was applied to collect information from patients or a member of their family regarding indirect and direct medical and non-medical costs. Catastrophic costs were calculated based on the proportion of patients with total costs exceeding 20% of their annual household income*.* The intensity of catastrophic costs was calculated using the positive overshoot method. The chi-square and Wilcoxon-Mann-Whitney tests were used to compare proportions and costs. Meanwhile, the Mantel Haenszel test was performed to assess the association between catastrophic costs and type of diagnosis.

**Results:**

Ninety-nine patients were interviewed (50 ACF and 49 PCF). Patients diagnosed through ACF incurred lower costs during the pre-treatment period (direct medical: USD 14 vs USD 32, *P* = 0.001; direct non-medical: USD 3 vs USD 10, *P* = 0.004; indirect, time loss: USD 4 vs USD 13, *P* <  0.001). The cost of the pre-treatment and intensive phases combined was also lower for direct medical (USD 15 vs USD 34, *P* = 0.002) and non-medical (USD 30 vs USD 54, *P* = 0.022) costs among ACF patients. The prevalence of catastrophic direct costs was lower for ACF patients for all thresholds. A lower intensity of catastrophic costs was also documented for ACF patients, although the difference was not statistically significant.

**Conclusions:**

ACF can reduce patient-incurred costs substantially, contributing to the End TB Strategy target. Other synergistic policies, such as social protection, will also need to be implemented to reduce catastrophic costs to zero among TB-affected households.

## Multilingual abstracts

Please see Additional file 1 for translations of the abstract into the five official working languages of the United Nations.

## Background

The World Health Organization (WHO) End TB Strategy has established a goal to end the global tuberculosis (TB) epidemic. A key milestone to be achieved by 2020 is reducing the number of TB-affected households facing catastrophic costs to zero [1]. A recent systematic review including studies of sufficient quality with low risk of bias conducted in Nigeria, Peru, China, and Moldova analyzed the effect of cash interventions on treatment outcomes. The review concluded that patients receiving a TB-specific cash transfer were more likely to have a positive clinical outcome than patients in the control group (odds ratio [*OR*]: 1.77; 95% confidence interval [*CI*]: 1.57–2.01) [2]. However, cash transfers alone are unlikely to eliminate catastrophic costs. Active case finding (ACF) has been recommended by international agencies as a supportive strategy to reduce the financial burden faced by TB patients [3, 4].

Studies have shown the importance of scaling up ACF to eliminate the gap between estimated and notified TB cases. The degree of case finding within national TB programs varies globally and therefore ACF interventions may encompass a range of strategies depending on the underlying context. These can include: household or social contact tracing, door-to-door screening, or targeted screening of high-risk groups.

In Nepal, the implementation of ACF by the Birat Nepal Medical Trust (BNMT) under the STOP TB/TB REACH funding programme Wave 2 (ref) was conducted in 15 Nepalese districts and detected 968 additional cases in 18 months (from January 2013 to June 2014) [5]. The ACF in Tuberculosis Trial (ACT2), which analyzed the impact of ACF using a household contact investigation of TB detection in Viet Nam, showed that the implementation of ACF, in addition to strong passive case finding (PCF), increased TB case detection from 703 per 100 000 population in the control districts to 1788 per 100 000 population in the intervention districts. Intensive household contact tracing was also found to reduce all-cause mortality in the intervention districts from 1.7% (control districts) to 0.6% (intervention districts; relative risk: 0.60; 95% *CI*: 0.50–0.80; *P* <  0.001) [6]. The analysis found that household contact tracing is a highly cost-effective intervention when compared with PCF alone (USD 544 per disability-adjusted life year averted) [7].

The implementation of ACF through TB REACH has also dramatically increased the number of cases detected in Ethiopia [8] and Cambodia, in the context of extremely weak underlying national TB programs [9]. However, increases in overall notification at the national level have not been shown through these small-scale, short-term projects, thus justification for national TB programs and global funders to invest in ACF remains weak. The Zambia South Africa Tuberculosis and HIV/AIDS Reduction (ZAMSTAR) cluster randomized trial of enhanced TB case finding in the context of a high HIV prevalence failed to show an impact on culture-confirmed TB prevalence after 4 years of intervention (*OR* = 1.09, 95% *CI*: 0.86–1.40) [10]. Furthermore, there is a lack of data to determine whether ACF can reduce patient-incurred costs. The WHO has been advocating strongly for research evidence from diverse settings to inform policy development to achieve the milestone of zero catastrophic costs [11].

In Nepal, the expansion of ACF is a key part of the Strategic Interventions to Increase TB Case Notification [12]. The National TB Programme (NTP) has planned to expand ACF activities through the implementation of community TB screening camps, screening of household and social contacts of index TB patients, and scaling up of GeneXpert® MTB/RIF testing (Xpert) [13]. Nepal has continued to face challenges in crucial areas, such as a sustained case detection gap, a poorly functioning health system, and high dependence on international donor funding for health (45% of the total budget) [14, 15]. Furthermore, a significant proportion of TB patients seek care in the private sector due to weak public services [15], increasing the risk of financial hardship for the most vulnerable.

Previous cost and cost-effectiveness studies on TB conducted in Nepal have evaluated patient-incurred costs under either community-based or family member directly observed treatment strategies, short-course (DOTS) for TB control [16] and direct costs of outpatient visits to obtain a TB diagnosis [17, 18]. This is the first study to evaluate and compare patient costs incurred through a diagnosis via ACF and PCF in the country. In a scenario of scarce financial resources, health economic evaluations play a key role in supporting the rational allocation of resources and informing evidence-based policy development. Therefore, the objective of this study was to compare costs incurred by pulmonary TB patients diagnosed through ACF and PCF, and determine the difference in prevalence and intensity of catastrophic costs between these groups.

## Methods

### Setting

Nepal is a low-income country with a population of 29 million people and a gross domestic product of USD 689 per capita [19]. In 2010, the poverty headcount ratio indicated that 25% of the population was living below the national poverty line [19]. In 2017, 31 764 cases of TB were notified by the NTP. The estimated TB incidence rate was 152 cases per 100 000 inhabitants, giving a case notification gap of more than 12 000 cases per year [14].

This TB patient cost study was conducted in two districts of the BNMT TB REACH Wave 5 project in Nepal, which aimed to increase case notifications of TB through the implementation of ACF models (June 2017 – December 2018). The BNMT TB REACH project was implemented in eight districts, with four districts applying Xpert for diagnosis (Pyuthan, Bardiya, Kapilvastu, and Gulmi) and four districts using smear microscopy (Doti, Achham, Argakhachi, and Salyan). The ACF model adopted three strategic interventions to identify TB patients: (1) contact tracing of social contacts; (2) TB camps for remote populations; and (3) screening at outpatient departments (OPDs) of public hospitals (Additional files 2 and 3). Household contacts were not evaluated in this TB REACH study because this was being carried out in the project areas as part of The Global Fund activities of the NTP.

This TB patient cost survey was conducted in two districts where the Xpert intervention was implemented: Pyuthan and Bardiya, Province No. 5 (Additional file 4). Pyuthan is a hilly district covering an area of 1309 km^2^ and has a population of 228 102 inhabitants [20]. It is classified as a district with a medium TB burden by the NTP, with 285 cases registered in 2017 [21]. Bardiya is a lowland Terai district covering an area of 2025 km^2^ and has a population of 426 576 inhabitants [20]. The district is classified as having a high TB burden, with 601 cases registered by the NTP in 2017 [21]. There is one government hospital in each district. In Bardiya, there are 29 health posts and three primary healthcare centers. In Pyuthan, there are 44 health posts and two primary healthcare centers. During the TB REACH project, 16 and seven TB camps were held in Bardiya and Pyuthan districts, respectively.

### Study design and sampling

A cross-sectional study was conducted between June and August 2018. As no data from Nepal were available to inform a sample size determination, we set a sample size based upon a previous cost survey (TB FIT: *Filipino Impact Assessment of new tuberculosis diagnostics*)) [22], which was sufficient to demonstrate an effect.

One hundred patients were included in this study in a 1:1 ratio (PCF:ACF, 25 consecutive ACF and 25 consecutive PCF patients in each district). ACF patients who were between 2 weeks and 3 months into the intensive phase of TB treatment were selected from a study database of all patients diagnosed via ACF strategies. PCF patients were identified from the treatment registers at DOTS centers in each district. No eligible patients declined participation.

### Inclusion criteria

All adult (≥ 18 years) new and relapse TB cases registered in government facilities and who were residents of Nepal were eligible for inclusion.

### Case finding interventions

Three interventions were applied in the TB REACH project. Details of the interventions are given in a paper reporting the results of the ACF intervention (forthcoming), and are described briefly below.

For the contact tracing intervention, a registered list of TB patients (index patients) diagnosed between July 2016 and July 2017 was obtained from each government treatment facility. Community Health Workers (CHWs) contacted the index patients and interviewed them to identify their social contacts. With the consent of the index patient, identified social contacts were then contacted and screened for signs and symptoms of TB using a simple symptom questionnaire (presence of cough for more than 2 weeks, blood in cough, fever, night sweats, or weight loss). Individuals reporting any of these symptoms were then invited to provide a sputum sample for testing. The CHWs collected the sputum sample in the morning and delivered the sample for testing to the nearest diagnostic facility. Those with a positive result received counseling and were referred to initiate TB treatment at the nearest health facility. Symptomatic individuals in more remote areas of the district who tested negative by smear were referred for Xpert testing at the district hospital. CHWs followed up newly diagnosed TB patients to facilitate treatment registration.

The second intervention was the establishment of TB camps in high burden or remote areas of the districts to screen, test with Xpert, and diagnose TB patients. CHWs conducted door-to-door screening using the symptom questionnaire 1 or 2 days prior to the camps being set up. Patients diagnosed with TB were enrolled into treatment by the CHWs at the nearest TB treatment center.

Symptomatic screening at the government district hospitals’ OPDs was conducted among attendees using the symptom questionnaire. Symptomatic individuals were asked to provide a sputum sample for Xpert testing and those with a positive diagnosis were enrolled into treatment at the treatment center nearest to their place of residence. Rifampicin-resistant cases were referred to multi-drug-resistant (MDR) TB services.

### Time horizon

Costs were collected at one point in time during the intensive phase of treatment. The interviewers collected information regarding costs incurred during the pre-treatment period (that is, from the onset of the first reported TB symptom until the first visit to a health facility for initiating TB treatment) and during the intensive phase of treatment until the date of the interview. (that is, within 60 days of treatment initiation for new cases and 90 days for relapse cases). Costs incurred during the intensive phase were extrapolated according to the number of remaining days of treatment: costs incurred from treatment until the date of interview x the proportion of the intensive phase to be completed; for example, if a patient was interviewed on the 30th day (half of intensive phase completed for new cases), the cost incurred until the day of the interview was multiplied by two (proportion of intensive phase to be completed = 60/30) [23].

### Collecting data on costing

The WHO TB patient costing questionnaire was adapted for this study. The questionnaire included questions on clinical parameters; demographic variables; information on employment and household composition; socioeconomic position; healthcare utilization, including the number of visits and costs (direct medical and non-medical) incurred during each visit in all types of health institutions; time and income lost (indirect costs) while seeking and receiving care; individual and family income; coping mechanisms, such as loans taken, assets sold; and the financial and social impacts of TB on patients and families.

The questionnaire was translated into Nepali and was pre-tested on seven patients undergoing TB treatment in Bardiya and Pyuthan. Minor corrections to the Nepali version of the questionnaire were made following this pilot testing. CHWs were trained in informed consent procedures and to administer the interviews. They were allocated to areas where they had relationships of trust in the community. CHWs prepared a list of TB patients diagnosed through ACF and PCF during the intervention period and contacted them to schedule an interview at their home or at the health facility. Those diagnosed via household contract tracing in The Global Fund program were not included in either group because the study’s aim was to compare the TB REACH interventions with passive patient presentation. Eligible individuals were invited to participate, informed about the purpose of the study orally and by a written patient information sheet (PIS), and were given an opportunity to ask questions. The PIS was read to individuals with low literacy levels. Written informed consent was obtained, or a thumbprint for those unable to sign, following standard Nepali practice. Compensation of 500 Nepalese rupees (NPR) (approximately USD 4.5) was provided for the time taken to complete the questionnaire (approximately 90 min).

Data completeness and consistency of information were assessed after each interview and were cross-checked with the patient treatment card. Data quality control was performed by the district TB coordinators, a research associate, and the data manager.

### Data entry and analysis

The WHO definition was applied to estimate the proportion of TB-affected households experiencing catastrophic costs: that is, the total costs (direct plus indirect) of seeking TB diagnosis and care which exceeds 20% of the annual household income [23]. We calculated the prevalence (that is, the proportion of patients with total costs > 20% of annual household income) and the intensity of catastrophic costs (using the positive overshoot method; that is, the average degree by which catastrophic costs exceed the 20% threshold) [24] for each group. Income loss, and individual and household income were self-reported by patients. Time loss was also self-reported by patients and converted to monetary values using the human capital approach applying hourly and monthly minimum wages of USD 0.62 and USD 4.67, respectively [25]. Costs were collected in NPR and were converted to USD applying the average exchange rate from OANDA during the data collection period (NPR 1 = USD 0.00903) (https://www1.oanda.com/) [26].

Data were entered by a trained technician into a bespoke web tool hosted by Koninklijke Nederlandse Centrale Vereniging tot bestrijding der Tuberculose (KNCV) TB Foundation and BNMT. Data analysis was performed using Stata version 15 (StataCorp, College Station, Texas 77 845, USA). The mean imputation approach was used to handle missing data and missing values were replaced by the mean value of the costing items [27]. The patients in each study arm were compared on socioeconomic and clinical characteristics. The impact of ACF on costs was determined by analyzing: (1) income changes and social consequences of TB; (2) median costs per cost component: that is, direct medical costs (drugs, tests, consultation fees, hospitalization charges), non-medical direct costs (transportation, food, accommodation), and indirect costs (time and income loss); (3) median cost per period of analysis (that is, the pre-treatment and intensive phases); and (4) proportion of direct and indirect costs per period of analysis.

The chi-square test was applied to test the difference in proportions of categorical variables. The non-parametric Wilcoxon-Mann-Whitney test was used to compare continuous variables (that is, costs). The Mantel-Haenszel analysis was performed to assess if the association between catastrophic costs and type of diagnosis (ACF vs PCF) was modified by other variables (gender, age, disease category, poverty line, dissaving, financial and social impacts). Stratified and pooled *OR*s and 95% *CI*s were reported together with the *P*-value for the homogeneity test [28]. All *P*-values below 0.05 were considered statistically significant.

A sensitivity analysis was performed to assess the impact of varying the threshold for catastrophic costs (10, 20, 30, 40, 50, and 60%) on the prevalence of catastrophic costs for ACF and PCF patients. The prevalence of catastrophic costs was also calculated using only the total direct costs as a proportion of the household annual income.

The effect of recall bias was also assessed in both groups. Median and interquartile costs were calculated for ACF and PCF patients interviewed within 1 month and after 1 month of treatment initiation.

## Results

### Patient characteristics

One hundred consecutively diagnosed TB patients were recruited. One PCF patient with extrapulmonary TB was excluded, thus the final sample was 99 patients: 50 diagnosed through ACF (three, 30, and 17 diagnosed via TB camps, OPDs, and contact tracing, respectively) and 49 diagnosed through PCF. All patients were interviewed during the intensive phase, within 14 to 90 days of treatment initiation, with 38% of patients interviewed during the first month of treatment. All eligible patients invited to participate gave written informed consent. Although MDR patients were included in the eligibility criteria, there were no MDR TB patients among those recruited. This is consistent with the MDR TB prevalence of < 1% in these districts.

No differences in the socioeconomic characteristics were found when comparing ACF and PCF patients (see Table 1). The majority of patients were male (71%), consistent with the 2:1 ratio of males and females in national TB notification data. Twenty-five percent were aged over 65 years and 47% were farmers. The most common source of drinking water was piped (49%) and the majority had a standard toilet (latrine) in the home (74%). Electricity (86%), a mobile phone (87%), and a bed (87%) were the most frequent assets reported (see Table 1).

**Table 1:** Socio-economic characteristics of tuberculosis patients diagnosed through active case finding (ACF) and passive case finding (PCF), Nepal, 2018

Patient features	ACF *N* = 50	PCF *n* = 49	All *n* = 100	*P*-value^a^
	*n* (%)	*n* (%)	*n* (%)	
Sex				
Female	18 (36)	11 (22)	29 (29)	0.139
Male	32 (64)	38 (77)	70 (71)	
Age group				
15–24	7 (14)	5 (10)	12 (12)	0.367
25–34	3 (6)	10 (20)	13 (13)	
35–44	9 (18)	8 (16)	17 (17)	
45–54	10 (20)	8 (16)	18 (18)	
55–64	10 (20)	6 (12)	16 (16)	
65+	11 (22)	12 (24)	23 (26)	
Education status^b^				
No education or illiterate	14 (28)	18 (36)	32 (32)	0.536
Literate	12 (24)	8 (16)	20 (20)	
Basic schools	20 (40)	16 (32)	36 (36)	
Secondary schools	4 (8)	6 (12)	10 (10)	
Master’s	–	1 (2)	1 (1)	
Occupation				
Farmer	29 (58)	18 (36)	47 (47)	0.229
Homemaker	6 (12)	5 (10)	11 (11)	
Others	15 (30)	26 (53)	41 (41)	
Patient as main income earner				
Yes	19 (38)	17 (34)	36 (36)	0.677
Source of drinking water				
Piped	24 (48)	25 (50)	49 (49)	0.804
Well	1 (2)	2 (4)	3 (3)	
Other	25 (50)	23 (46)	48 (48)	
Toilet facilities				
No toilets	4 (8)	3 (6)	7 (7)	0.936
latrine	36 (72)	38 (76)	74 (74)	
Public sewage	4 (8)	3 (6)	7 (7)	
Septic tank	6 (12)	5 (10)	11 (11)	
Assets^c^				
Electricity	43 (86)	43 (86)	86 (86)	1
Radio	18 (36)	24 (48)	42 (42)	0.224
Mobile phone	42 (84)	45 (90)	87 (87)	0.372
Table	22 (44)	22 (44)	44 (44)	1
Chair	23 (46)	25 (50)	48 (48)	0.689
Bed	44 (88)	43 (86)	87 (87)	0.766
Cupboard	14 (28)	17 (34)	31 (31)	0.517
Clock	14 (28)	14 (28)	28 (28)	1
Fan	18 (36)	18 (36)	36 (36)	1
Watch	20 (40)	22 (44)	42 (42)	0.685
Bicycle	22 (44)	18 (36)	40 (40)	0.414
Television	16 (32)	19 (38)	35 (35)	0.529
Livestock, small	37 (74)	40 (80)	77 (77)	0.476
Livestock, large	31 (62)	28 (56)	59 (59)	0.542

^a^ Chi square

^b^ Literate = able to only read and write, Basic schools = primary level/lower secondary level (1 to 8 year of education)

^c^ Other assets: refrigerator, ACF – 2 (4) and PCF - 4 (8); sofa, ACF - 1 (2) and PCF - 2 (4); computer, ACF – 1 (2) and PCF – 2 (4); motorcycle, ACF 4 (8) - and PCF – 2 (4); Animal-drawn cart, ACF – 5 (10), PCF 3 (6); thresher, ACF 1 (2)

### Disease and treatment characteristics

No differences were documented in disease characteristics when comparing patients diagnosed by ACF or PCF. The majority of patients were classified as new TB cases (83%) and no patient reported a HIV positive status. A similar proportion of both groups (ACF vs PCF) visited private health services during the pre-treatment period (37% vs 41%) and sought diagnosis using public services (52% vs 54%). The average number of visits to health facilities during the pre-treatment period (2.3 vs 2.6) and the average number of follow-up visits after treatment initiation (0.2 vs 0.4) were lower among ACF patients. However, statistical significance was reached only for follow-up visits (*P* = 0.026). The average number of weeks between the first symptom and treatment initiation was similar for ACF and PCF patients (8.4 vs 8.8, *P* = 0.638) (see Table 2).

**Table 2:** Disease and treatment characteristics of tuberculosis patients diagnosed through active case finding (ACF) and passive case finding (PCF), Nepal, 2018

Characteristics	ACF *n* = 50	PCF *n* = 49	All *n* = 100	*P*-value ^a^
Treatment status	*n* (%)	*n* (%)	*n* (%)	
New	42 (84)	40 (82)	82 (82)	0.755
Retreatment/Relapse	8 (16)	9 (18)	17 (17)	
HIV Status ^b^				
Not tested	8 (16)	4 (8)	12 (12)	0.485
Negative	39 (78)	40 (83)	79 (79)	
Unknown	3 (6)	4 (8)	7 (7)	
Number of patients with reported hospitalization				
Pre-treatment	4 (8)	10 (20)	14 (14)	0.099
Intensive phase	0	1 (2)	1 (1)	–
Total number of visits to health providers, pre-diagnosis ^c^	*N* = 114	*N* = 133	*N* = 247	–
Type of service visited, pre-diagnosis				
TB camps	8 (7)	0	8 (3)	0.031
Cross border service ^d^	0	1 (1)	1 (0.5)	
Pharmacy/Herbalist	5 (4)	5 (4)	10 (4)	
Private clinic/hospital	42 (37)	55 (41)	97 (39)	
Public health facility	59 (52)	72 (54)	131 (53)	
Total number of visits to health providers, intensive phase ^c^	*N* = 61	*N* = 64	*N* = 125	–
Type of service visited, intensive phase				
No health facility	14 (23)	21 (33)	35 (28)	0.102
Private clinic/hospital	7 (11)	13 (20)	20 (16)	
Public health facility	40 (66)	30 (47)	70 (56)	
Average number of visits to health providers	Mean (SD)	Mean (SD)	Mean (SD)	
Health facility visits – pre-treatment	2.3 (1.1)	2.6 (1.6)	2.5 (1.4)	0.380
Health facility visits – intensive phase	0.9 (0.9)	0.9 (0.9)^c^	0.9 (0.9)	0.500
Follow-up visits - Intensive phase	0.2 (0.6)	0.4 (1.0)	0.3 (0.7)	0.026
Average number of days hospitalised				
Pre-treatment	5.3 (3.8)	8.1 (7.0)	7.5 (6.4)	0.638
Average number of weeks between 1st TB symptoms and treatment initiation	8.4 (8.0)	8.8 (11.3)	8.6 (9.8)	0.931

^a^ Two-sample Wilcoxon rank-sum (Mann-Whitney) test and Chi square

^b^ There is one missing data in HIV status

^c^ Patients can have more than one visit to different health facilities

^d^ Patient crossed the border to visit a health facility in India

### Income changes and social consequences

PCF patients reported a higher economic impact due to TB treatment when compared with ACF patients, with 20% of PCF patients declaring being much poorer after TB treatment initiation, while among ACF patients this proportion was 2% (*P* = 0.016). TB resulted in a substantial decrease in the individual and household incomes of individuals diagnosed by either ACF or PCF. However, the higher impoverishment rate among PCF patients did not appear to be a consequence of income reduction: there was no difference in the income reduction between the diagnostic groups, but rather time loss and out-of-pocket expenses (further details below). The individual income reduced by 75 and 74% for ACF and PCF patients, respectively. The reduction in the household income was 37 and 38% for ACF and PCF patients, respectively. The poverty headcount during the intensive treatment phase also increased substantially in both diagnostic groups: 160 and 167% for individuals diagnosed by ACF or PCF, respectively. A quarter of all patients (26%) reported food insecurity as a consequence of TB (see Table 3).

**Table 3:** Income changes and social consequences of tuberculosis in patients diagnosed through active case finding (ACF) and passive case finding (PCF), Nepal, 2018

Item	ACF *n* = 50	PCF *n* = 49	All *n* = 100	*P*-value ^a^
Income (USD)	Mean (SD)	Mean (SD)	Mean (SD)	
Individual income prior TB	79 (88)	70 (83)	80 (85)	0.602
Household income prior TB	196 (111)	182 (184)	189 (151)	0.052
Current individual income^b^	20 (44)	18 (37)	19 (40)	0.951
Current household income^b^	123 (101)	113 (174)	118 (142)	0.080
Working hours per week				
Prior TB	31 (28)	29 (29)	30 (29)	0. 584
Current^b^	5 (11)	4 (11)	4 (11)	0. 643
Catastrophic costs				
Intensity ^c^	61 (53)	88 (172)	76 (132)	0.6713
	N (%)	N (%)	N (%)	
Prevalence ^d^	20 (45)	24 (61)	44 (53)	0.143
Employment status prior TB				
Unemployed	2 (4)	5 (10)	7 (7)	0.475
Formal paid work	4 (8)	7 (14)	11 (11)	
Informal paid work	24 (48)	17 (34)	41 (41)	
Housework	15 (30)	13 (26)	28 (28)	
Others	5 (10)	8 (16)	13 (13)	
Current employment status ^b^				
Unemployed	13 (26)	18 (36)	31 (31)	0.310
Formal paid work	–	2 (4)	2 (2)	
Informal paid work	5 (10)	2 (4)	7 (7)	
Housework	29 (58)	23 (46)	52 (52)	
Others	3 (6)	5 (10)	8 (8)	
Poverty headcount ^e^				
Before TB	5 (10)	6 (12)	11 (11)	0.749
Current^b^	13 (26)	16 (32)	29 (29)	0.509
Dissaving strategies ^b^				
Loan	14 (28)	22 (44)	36 (36)	0.096
Sale of assets	4 (8)	5 (10)	9 (9)	0.727
Social impact ^b^				
Food insecurity	13 (26)	13 (26)	26 (26)	1
Loss job	2 (4)	4 (8)	6 (6)	0.400
Interrupted schooling	4 (8)	2 (4)	6 (6)	0.400
Social exclusion	10 (20)	7 (14)	17 (17)	0.424
Others	4 (8)	1 (2)	5 (5)	0.169
Financial impact^b^				
Much poorer	1 (2)	10 (20)	11 (11)	0.016
Poorer	26 (52)	22 (44)	48 (48)	
Unchanged	23 (46)	18 (36)	41 (41)	

^a^ Chi square

^b^ Intensive phase

^c^ Intensity of catastrophic costs measured as median-positive overshoot beyond the 20% threshold

^d^ Percentage of patients with total costs > 20% of annual family income (WHO)

^e^ Number of families living with an annual income per capita below NPR 12000 (2011 prices) (http://www.thepovertyline.net/nepal

### Costs

For the pre-treatment period, ACF patients reported lower direct medical (USD 14 vs USD 32; *P* = 0.001), non-medical (USD 3 vs USD 10; *P* = 0.004), and indirect (USD 4 vs USD 13; *P* <  0.001) costs, the latter measured using the human capital approach (that is, based on time loss). The median total costs in this phase were also lower for ACF patients, although not statistically significant (USD 132 vs USD 172, *P* = 0.103) (see Table 4).

**Table 4:** Median pre-treatment and treatment costs in tuberculosis patients diagnosed through active case finding (ACF) and passive case finding (PCF), Nepal, 2018

Cost item	ACF (*n* = 50)	PCF (*n* = 49)	Total (*n* = 100)	*P*-value^b^
Pre-treatment period	Median (IQR)	Median (IQR)	Median (IQR)	
Direct medical				
Consultation fee	0.0 (0.0–0.1)	0.2 (0.0–4.5)	0.0 (0.0–1.0)	0.003
Radiography	0.5 (0.0–3.2)	3.2 (0.0–9.9)	1.6 (0.0–5.6)	0.003
Lab tests	1.1 (0.0–3.4)	2.7 (0.0–5.7)	1.8 (0.0–4.1)	0.092
Medicines	5.9 (0.0–16.8)	18.3 (1.5–36.7)	8.1 (0.5–25.6)	0.021
Other medical	0.0 (1.8–4.5)	3.3 (1.4–6.6)	2.7 (0.0–5.8)	0.013
Total direct medical	14.3 (4.5–27.7)	31.6 (11.0–79.1)	19.2 (6.3–46.3)	0.001
Direct non-medical				
Transportation	3.3 (0.9–7.2)	5.4 (1.8–15.5)	3.7 (1.8–10.4)	0.031
Food	–	0.0 (0.0–10.8)	0.0 (0.0–2.7)	0.006
Total direct non-medical	3.4 (1.8–10.4)	9.7 (2.7–37.9)	5.4 (2.1–22.4)	0.004
Indirect				
Time loss^a^	4.4 (1.9–8.1)	13.4 (5.6–21.8)	7.8 (3.7–15.0)	< 0.001
Income loss	51.4 (0.0–240.1)	30.7 (0.0–201.8)	40.6 (0.0–212.9)	0.629
Total indirect	63.5 (5.0–255.1)	43.3 (14.3–248.2)	51.1 (8.4–251.6)	0.430
*Total cost pre-treatment*	*132.3 (22.6–258.0)*	*172.3 (59.9–405.4)*	*147.3 (41.6–304.9)*	*0.103*
Intensive phase				
Direct medical				
Consultation fee/charges	–	–	–	–
Radiography/lab tests	–	–	–	–
Medicines	–	0.0 (0.0–1.8)	–	0.045
Total direct medical	–	0.0 (0.0–4.0)	–	0.070
Direct non-medical				
Transportation	0.0 (0.0–7.2)	0.4 (0.0–17.3)	0.0 (0.0–8.5)	0.041
Food	0.0 (0.0–6.8)	0.0 (0.0–19.5)	0.0 (0.0–7.7)	0.547
Total direct non-medical	0.0 (0.0–14.4)	1.3 (0.0–44.8)	0.0 (0.0–28.0)	0.034
Indirect				
Time loss^a^	29.9 (15.0–44.9)	31.0 (11.7–59.8)	29.9 (15.0–56.1)	0.816
Income loss	18.1 (0.0–49.7)	9.6 (0.0–45.2)	17.1 (0.0–45.2)	0.377
Total indirect	54.9 (29.9–95.9)	59.6 (34.9–82.7)	55.1 (29.9–90.5)	0.817
Other				
Nutritional supplements	13.6 (7.5–25.4)	15.5 (9.3–35.3)	14.9 (8.1–27.8)	0.404
*Total costs intensive phase*	*84.7 (56.1–144.0)*	*103.7 (45.3–193.2)*	*96.6 (51.8–176.9)*	*0.557*
Total cost pre-treatment and intensive phase				
Total direct medical costs	14.9 (4.5–46.1)	34.1 (13.1–87.5)	22.6 (6.7–63.8)	0.002
Total direct non-medical costs	29.6 (15.6–55.1)	54.0 (21.5–124.6)	37.5 (17.8–83.5)	0.022
Total direct costs	40.2 (26.1–91.7)	114.9 (45.3–250.5)	68.5 (31.7–148.6)	0.001
Total indirect costs	128.2 (34.9–357.4)	106.1 (57.8–340.9)	112.4 (52.4–343.7)	0.942
*Total* ^*c*^	*252.8 (80.9–452.8)*	*315.3 (125.8–543.9)*	*290.1 (88.7–476.7)*	*0.161*

^a^ Hourly minimum wage: USD 0.62; Daily minimum wage: USD 4.67 (http://www.pioneerlaw.com/news/minimum-wage-remuneration-2018–2075)

^b^ Wilcoxon-Mann-Whitney

^c^ Total cost: from the 1st TB symptoms until the end of intensive phase

During the intensive treatment phase, ACF patients also incurred lower direct non-medical (USD 0 vs USD 1), indirect (USD 55 vs USD 60), and total (USD 85 vs USD 104) costs. However, statistical significance was found only for direct non-medical costs (*P* = 0.034).

The median total cost (pre-treatment plus intensive phase) was also lower for ACF patients, particularly for direct medical (USD 15 vs USD 34, *P* = 0.002) and non-medical (USD 30 vs USD 54, *P* = 0.022) costs. The total direct costs were 65% lower for ACF patients compared with PCF patients (USD 40 vs USD 115, *P* = 0.001) (see Table 4).

**Fig. 1:**
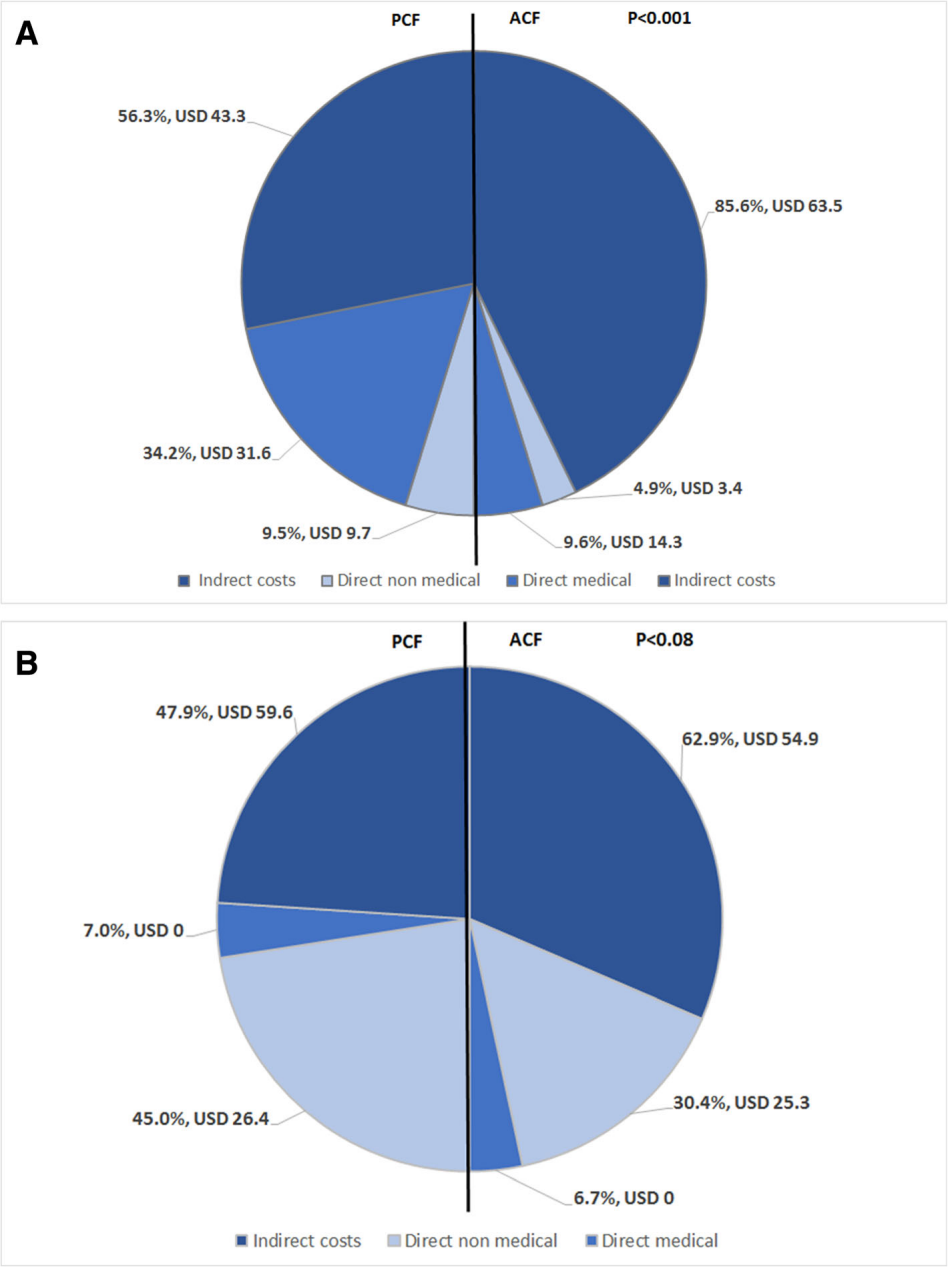
The proportion of total costs attributed to each cost category (indirect, direct medical and direct non-medical) for patients diagnosed by either ACF (right side of pie chart) or PCF (left side of pie chart). The median cost incurred for each category is also shown in USD. *Legend: P*-value: Pearson’s chi-square test

Indirect costs, particularly income loss, were the main driver of the total costs for both groups during the pre-treatment and intensive phases. However, PCF patients had higher percentages of direct medical (34% vs 10%) and non-medical (9% vs 5%, *P* <  0.001) costs during the pre-treatment period (see Fig. 1).

### Catastrophic costs

Eighty-four patients were included in this analysis as 15 patients were unable to report the value of household income. ACF patients presented 26% lower prevalence (45% vs 61%) and 69% lower intensity (53% vs 172%) of catastrophic costs, considering direct and indirect costs (see Table 3).

Stratified analysis (Mantel-Haenszel) used to investigate variables influencing the association of diagnostic strategy with risk of catastrophic costs showed that stratification by gender, TB relapse, poverty level, dissaving, and financial and social impacts did not change the *OR* of incurring catastrophic costs. However, stratification by age revealed significant heterogeneity in the odds of incurring catastrophic costs (*P* = 0.043), with those aged under 60 years having an *OR* of 4.6 (95% *CI*: 1.19–19.32) for catastrophic costs when diagnosed passively rather than actively, compared to an *OR* of 0.6 (95% *CI*: 0.93–3.61) in those aged over 60 years (see Table 5).

**Table 5:** Association between catastrophic costs and passive case finding (PCF)/ active case finding (ACF) adjusted for each exposure variable at time, Nepal, 2018

Variable	Cases of catastrophic costs per diagnostic method *n*/*N* (%) ^a^		*OR*^b^ (95% confidence interval)		*P*-value ^c^
	PCF *N* = 45	ACF *N* = 39	Stratified	Pooled	
*Total population*	24/39 (61)	20/45 (44)	2 (0.77–5.25)		
Variables					
Gender					
Male	20/31 (65)	14/28 (50)	1.8 (0.56–5.89)	1.8 (0.75–4.44)	0.993
Female	4/8 (50)	6/17 (35)	1.8 (0.24–13.84)		
Age					
< 60	20/25 (80)	14/30 (47)	4.6 (1.19–19.32)	2.1 (0.86–5.17)	0.043
≥ 60	4/14 (29)	6/15 (40)	0.6 (0.93–3.61)		
Disease category					
New case	19/33 (58)	17/38 (45)	1.7 (0.59–4.78)	2.0 (0.83–4.78)	0.330
Relapse	5/6 (83)	3/7 (43)	6.7 (0.34–392.48)		
Poverty line					
Bellow	4/5 (80)	5/8 (62)	2.4 (0.11–156.99)	2.1 (0.87–5.19)	0.924
Above	20/34 (58)	15/37 (40)	2.1 (0.73–6.03)		
Dissaving					
Yes	13/18 (72)	9/15 (60)	1.7 (0.32–9.60)	1.8 (0.75–4.49)	0.922
No	11/21 (52)	11/30 (37)	1.9 (0.53–6.84)		
Financial impact					
Poorer /Much poorer	15/22 (68)	14/26 (54)	1.8 (0.48–7.15)	2.1 (0.85–5.06)	0.758
Unchanged	9/17 (53)	6/19 (31)	2.4 (0.52–11.78)		
Social impact^d^					
Yes	10/14 (71)	14/23 (61)	1.6 (0.32–9.13)	2.5 (0.98–6.23)	0.437
No	14/25 (56)	6/22 (27)	3.4 (0.86–14.08)		

^a^ Five ACF and 11 PCF patients with “zero” annual family income excluded from this analysis

^b^ Odds ratio (*OR*) was calculated using Mantel-Haenszel method

^c^ *P*-value is from homogeneity test in Mantel-Haenszel analysis

^d^ Social impact: divorce or social exclusion or food insecurity or loss of job or Interrupted schooling

### Sensitivity analysis

**Fig. 2:**
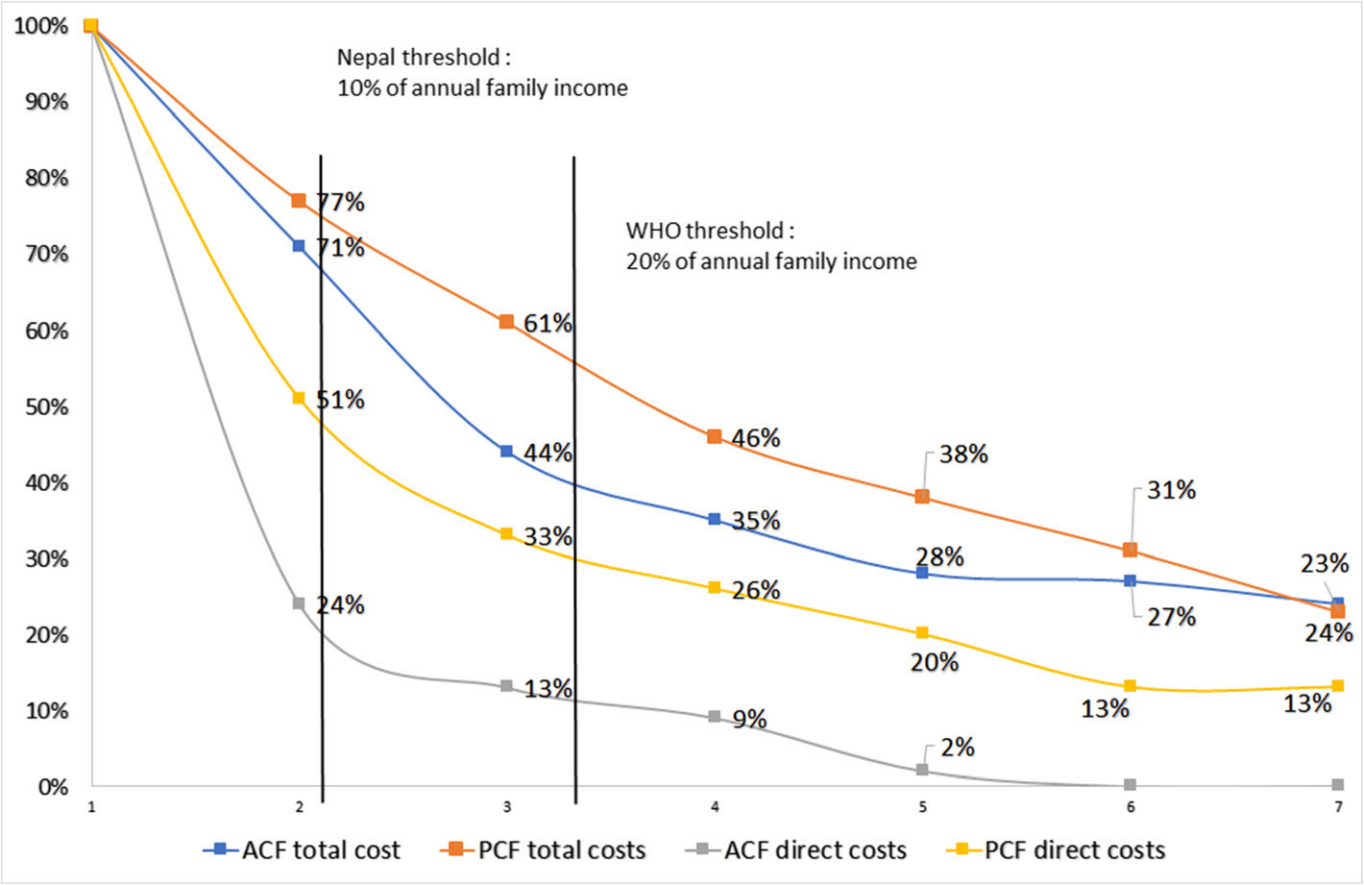
Prevalence of catastrophic costs in tuberculosis patients diagnosed through active case finding (ACF) and passive case finding (PCF) during the pre-treatment and intensive phases, Nepal, 2018

The prevalence of catastrophic costs was higher for PCF patients in all thresholds analyzed. Using the WHO threshold (that is, 20% of annual household income) and only direct costs, the prevalence of catastrophic costs was 61% lower for ACF patients when compared with PCF patients (13% vs 33%, *P* = 0.029) (see Fig. 2).

PCF patients were more strongly affected by recall bias and lower costs were reported for patients interviewed after 1 month of treatment initiation when compared with those interviewed within 1 month of treatment initiation. This suggests that in fact the PCF costs are more likely to be underestimated and the effect of ACF on reducing patient incurred costs is in fact greater than estimated from these self-reported data. PCF patients interviewed after 1 month of treatment reported lower indirect (USD 29 vs USD 282, *P* < 0.001) and total (USD 128 vs USD 366, *P* = 0.007) costs during the pre-treatment period, than PCF patients patient interviewed greater than 30 days after treatment initiation;. This difference was also seen for lower direct non-medical (USD 16 vs USD 81, *P* = 0.005) and total (USD 68 vs USD 190, *P* = 0.004) costs during the intensive phase; and lower indirect (USD 76 vs USD 367, *P* = 0.003) and total (USD 232 vs USD 556, *P* = 0.002) costs during both periods combined. There was no difference in costs among ACF patients interviewed within and after 1 month of treatment initiation (see Table 6).

**Table 6:** Median costs of patients interviewed within and after one month of treatment initiation, Nepal, 2018

Cost item	Active case finding		*P*-value^a^	Passive case finding		*P*-value^a^	Total		*P*-value^a^
	< 1 month (*n* = 22)	> 1 month (*n* = 28)		< 1 month (*n* = 16)	> 1 month (*n* = 34)		< 1 month (*n* = 38)	> 1 month (*n* = 62)	
	Median (IQR)	Median (IQR)		Median (IQR)	Median (IQR)		Median (IQR)	Median (IQR)	
Pre-treatment									
Direct medical	14.0 (0.0–27.7)	14.9 (6.3–31.7)	0.427	32.5 (8.6–80.7)	31.6 (14.3–79.1)	1	16.6 (4.3–46.1)	19.9 (9.6–46.5)	0.306
Direct non-medical	3.7 (2.7–7.2)	3.4 (1.4–12.1)	0.953	13.5 (2.3–49.3)	9.7 (2.7–35.2)	0.992	5.4 (2.7–21.7)	6.1 (2.0–22.6)	0.725
Indirect	51.0 (4.4–160.5)	68.1 (5.4–294.3)	0.814	281.8 (55.3–513.1)	28.8 (11.6–143.7)	< 0.001	99.2 (6.3–408.4)	36.0 (8.7–194.3)	0.088
*Total*	*121.8 (15.4–240.0)*	*146.7 (29.2–325.0)*	*0.639*	*365.9 (164.9–592.0)*	*128.5 (55.1–220.2)*	*0.007*	*121.8 (15.4–240.0)*	*146.7 (29.2–325.0)*	*0.237*
Intensive phase									
Direct medical	0	0	0.716	0	0	0.716	0	0	0.379
Direct non-medical	26.4 (14.1–45.9)	22.2 (8.4–37.0)	0.187	81.4 (33.8–141.8)	15.8 (6.5–38.6)	0.005	36.1 (18.1–78.0)	18.4 (8.1–37.8)	0.004
Indirect	51.5 (29.9–98.9)	54.9 (22.0–85.6)	0.494	63.5 (44.2–102.6)	54.2 (19.5–82.7)	0.126	58.8 (38.9–100.3)	54.9 (19.5–82.7)	0.128
*Total*	*113.3 (56.4–144.0)*	*72.7 (46.1–133.6)*	*0.282*	*190.4 (113.2–234.5)*	*67.6 (39.3–143.4)*	*0.004*	*122.8 (72.1–193.2)*	*71.6 (41.1–143.4)*	*0.005*
Total cost pre-treatment and intensive phase									
Direct medical	14.9 (0.0–33.3)	14.9 (6.3–46.3)	0.617	40.3 (9.6–95.8)	33.5 (16.9–87.5)	0.934	24.9 (4.5–67.0)	21.1 (9.6–59.6)	0.477
Direct non-medical	33.2 (18.2–33.2)	28.0 (11.9–55.2)	0.369	102.2 (36.1–195.7)	30.8 (19.3–95.0)	0.061	48.9 (20.8–99.8)	30.0 (15.4–73.3)	0.113
Indirect	105.7 (34.9–313.8)	163.6 (38.2–394.8)	0.953	366.9 (97.2–628.9)	76.3 (50.9–246.9)	0.003	165.7 (82.8–538.8)	90.2 (46.1–308.3)	0.039
*Total*	*209.3 (96.4–355.6)*	*280.5 (80.2–476.7)*	*0.815*	*556.3 (331.3–717.8)*	*232.3 (85.0–448.3)*	*0.002*	*337.5 (163.8–608.2)*	*244.4 (84.4–461.1)*	*0.091*

^a^ Chi square test

## Discussion

This study demonstrated that patients diagnosed through ACF incurred substantially lower costs than those diagnosed by PCF, with 65% lower direct costs and 61% lower catastrophic cost prevalence when considering only direct costs. The study also confirms the devastating financial impact of TB on poor households in Nepal and the high prevalence of catastrophic costs incurred by TB-affected households in both groups, but particularly among patients diagnosed by PCF who are aged under 60 years.

Other costing surveys conducted in Asia have also found lower costs and catastrophic costs among patients diagnosed through ACF when compared with PCF. In Cambodia, ACF patients incurred 79% lower total costs during the pre-treatment period (USD 5 vs USD 24, *P* < 0.001, costs inflated to 2018 prices) [29]. In India, a TB patient cost survey conducted in vulnerable populations found 75% lower total costs (USD 5 vs USD 20, *P* < 0.001, 2018 prices) and 32% lower catastrophic costs (adjusted prevalence ratio: 0.68, 95% *CI*: [0.69–0.97]) for ACF patients [30].

The findings of this study indicate that ACF has the potential to avert a substantial portion of direct costs and catastrophic direct costs associated with TB diagnosis and care, and can thereby help reduce the broader socioeconomic consequences of TB in Nepal. Previous TB patient cost surveys conducted among PCF patients in the country have found that high direct costs (that is, transportation, clinical fees, and tests) pose a barrier for patients seeking TB diagnosis and treatment [17, 18]. In addition, high costs have been associated with adverse TB outcomes such as a delay in seeking diagnosis and starting treatment [31, 32], death, and treatment abandonment or treatment failure [33]. Thus, the implementation of ACF can potentially contribute to improved treatment outcomes and reduce mortality [7]. These outcomes will be analyzed in an ongoing project in Nepal (IMPACT TB).

The impact of ACF on direct costs, particularly during the pre-treatment period, is principally a consequence of savings incurred in transportation and diagnostic tests. Nepal has a poor transport infrastructure, and many patients live in areas without roads and therefore have to travel several hours or even several days to reach a health service. ACF reduces or removes the need for patients to travel long distances to reach diagnostic centers, or make use of private health services, and pay for laboratory tests or radiography. ACF patients receive visits from healthcare workers for TB screening, sputum collection, and further referral for TB treatment for those with a positive diagnosis. Besides decreasing patient costs, ACF increases accessibility to health care.

Other community-based initiatives covering different areas of public health have been successful in improving access to health care. China’s barefoot doctor system (1968–1985) expanded the coverage of healthcare services, reduced costs, and provided timely treatment by training indigenous paramedics in rural areas of China [34]. In Nepal, female CHVs have also improved access to health care in urban and rural areas by delivering health promotion and prevention activities at the household level [35]. Furthermore, village health workers, who were focused on immunization programs, were promoted to auxiliary health workers by the Ministry of Health in 2014–2015. The new role was expanded to provide preventive and promotive health services and basic curative services for the community [35, 36].

A difference in total income loss was not identified in this study, probably because the ACF strategy did not appear to provide an earlier TB diagnosis. Earlier diagnosis among patients diagnosed through ACF was identified in previous studies in Cambodia [37] and Viet Nam [6], however, both studies evaluated ACF among household contacts of index patients in addition to social contacts.

This study has a number of limitations. First, the calculation of catastrophic costs considered self-reported household income. This approach does not consider dissaving strategies and it is more challenging to apply in countries with strong informal economies and seasonal fluctuations in income, such as Nepal. However, the interviewers were advised to ask about and explore the average annual monthly income, regardless of seasonality of the market. In addition, this approach has been widely used [29, 30, 33, 38], which allows for comparisons to be made between our findings and other studies. Second, the calculation of catastrophic costs did not include costs incurred during the continuation phase of treatment, thus its prevalence was underestimated. Third, the analysis did not detect an association between key variables, such as poverty line, social and financial impacts, dissaving strategies, and the occurrence of catastrophic costs. A larger sample size may be required to identify these associations. Fourth, patients were recruited at different time points during the intensive phase, which influenced the degree of recall bias [39]. In this study, PCF patients were more affected by recall bias than ACF patients, and may have underestimated their indirect, non-medical, and total costs as they were interviewed 1 month after treatment initiation. Thus, as these patients underestimated costs, the difference in costs between ACF and PCF patients may be even higher.

The study also has a number of strengths. Interviews were conducted by trained health workers who had a previous relationship with the community. The adoption of this strategy was crucial to collect complete and accurate data because a relationship of trust between interviewer and participant is essential when asking sensitive questions about personal or household income. The present study provides important evidence to inform policy evolution for ACF scale-up in Nepal. Knowledge of the components, drivers, and distribution of costs for TB-affected households will be necessary to develop and advocate for effective interventions to mitigate costs and achieve the End TB Strategy’s goal to reduce the number of TB-affected households facing catastrophic costs to zero. Our findings indicate that ACF is an important strategy to contribute to the achievement of this goal. A national TB patient cost survey for Nepal would provide comprehensive data and should be prioritized. The impact of ACF on catastrophic costs in other countries and population groups should also be robustly evaluated to inform global policy. Even though ACF reduced costs, the prevalence of catastrophic costs was still found to be very high in both groups. The expansion in coverage of social protection would play an important role in alleviating extreme poverty and, indirectly, in reducing TB incidence [40]. Cash transfer programs, such as Bolsa FamÍlia in Brazil, have been successful in reducing poverty and improving TB treatment outcomes [41]. In Peru, socioeconomic support for TB patients has improved TB outcomes and prevented catastrophic costs [33, 42]. Similar interventions should be piloted, evaluated, and integrated into the NTP in Nepal.

## Conclusions

ACF is an important strategy to avert direct costs and to reduce the proportion of TB households incurring catastrophic direct costs. Other policies, such as social protection, should be implemented in combination with ACF to mitigate the financial burden of TB, particularly among the most vulnerable populations.

## Supplementary information

**Additional file 1.** Multilingual abstracts in the five official working languages of the United Nations.

**Additional file 2.** Algorithm for TB REACH active case finding through contact tracing, Nepal, 2018.

**Additional file 3.** Algorithm for TB REACH active case finding through TB camps and outpatient department of public hospitals, Nepal, 2018.

**Additional file 4.** TB REACH districts coverage and costing survey districts, Nepal, 2018.

## Data Availability

The datasets generated and analyzed during the current study are not publicly available due to data protection law.

## Abbreviations

ACF: Active case finding
BNMT: Birat Nepal Medical Trust
CHWs: Community Health Workers
*CI*: Confidence interval
DOTS: Directly observed treatment, short-course
MDR: Multi-drug-resistant
NPR: Nepalese rupees
NTP: National TB Programme
OPD: Outpatient department
*OR*: Odds ratio
PCF: Passive case finding
PIS: Patient information sheet
TB: Tuberculosis
WHO: World Health Organization
