# Supplementary material for: The role of active case finding in reducing patient incurred catastrophic costs for tuberculosis in Nepal
Source: Infect Dis Poverty. 2019 Dec 3;8:99. doi: 10.1186/s40249-019-0603-z (PMC6889665; doi:10.1186/s40249-019-0603-z)
Supplement: Supplementary file 1 — Additional file 1. Multilingual abstracts in the five official working languages of the United Nations. [file 40249_2019_603_MOESM1_ESM.pdf]

Translation of the abstract into the five official working languages of the United Nations

دور الكشف النشط عن الحالات في الحد من التكاليف الكارثية التي يتحملها مرضى السل في نيبال

سومان تشاندرا غورونغ، وكريتيكا ديكسيت، وبولا راي، وماكسين كاوز، وبوسكار راج بودل، وراجو ديتال، وشرادها أتشاريا، وغانغارام بوداثوكي، وديباك مالا، وينس دبليو ليفي، ووب فان ريست، وكنوت لونروث، وكيري فيني، وأندرو رامساي، وتوم وينجفيلد بوذا باسنيات، وأنيل ثابا، وبيرتي سكوير، ودوولاو وانغ، وجوكول ميشرا، وكاشم شاه، وأنيل شريستا، ونيميا تيكسيرا دي سيكويرا فيلها

الملخص

الخلفية: حددت استراتيجية منظمة الصحة العالمية لمكافحة السل نقطة مرحلية لتقليل عدد الأسر المتأثرة بالسل التي تواجه تكاليف كارثية إلى الصفر بحلول عام 2020. لم يتم تحديد دور الكشف للحالات في تقليل تكاليف المرضى على مستوى العالم. تهدف هذه الدراسة بالتالي إلى مقارنة التكاليف التي تكبدها مرضى السل الذين تم تشخيصهم من خلال الكشف النشط للحالات والكشف غير النشط للحالات، وتحديد مدى انتشار وشدة التكاليف الكارثية التي تكبدها المريض في نيبال.

طرق: أجريت الدراسة في منطقتين في نيبال: بارديا وبيوثان (المقاطعة رقم 5) بين يونيو وأغسطس 2018. تم تضمين مائة مريض في هذه الدراسة بنسبة 1 :1 (كشف غير نشط عن الحالات:كشف نشط عن الحالات، 25 مريض كشف نشط عن الحالات متتالي و 25 كشف غير نشط عن الإحالات على التوالي في كل منطقة). تم تطبيق أداة تقدير تكاليف مرضى السل الخاصة بمنظمة الصحة العالمية لجمع المعلومات من المرضى أو أحد أفراد أسرهم بشأن التكاليف الطبية وغير الطبية المباشرة وغير المباشرة. تم حساب التكاليف الكارثية على أساس نسبة المرضى الذين تتجاوز تكاليفهم الإجمالية 20٪ من دخل الأسرة السنوي. تم حساب شدة التكاليف الكارثية باستخدام طريقة التجاوز الوضعي. استخدمت اختبارات مربع تشي ولوكسون مان ويتني لمقارنة النسب والتكاليف. في هذه الأثناء، تم إجراء اختبار مانتيل هاينسزل لتقييم الارتباط بين التكاليف الكارثية ونوع التشخيص.

النتائج: أجريت مقابلات مع تسعة وتسعين (50 كشف نشط عن الحالات و 49 كشف غير نشط عن الحالات). تكبد المرضى الذين تم تشخيصهم من خلال الكشف النشط عن الحالات تكاليف أقل خلال فترة ما قبل العلاج (طبية مباشرة: 14 دولارًا أمريكيًا) مقابل 32 دولارًا أمريكيًا، نسبة = 0.001 ؛ غير طبي مباشر: 3 دولارات مقابل 10 دولارات أمريكية، نسبة = 0.004 ؛ غير مباشر، ضياع الوقت: 4 دولارات أمريكية مقابل 13 دولارًا أمريكيًا، نسبة <0.001). كانت تكلفة المراحل الأولية والعلاج المكثف مجتمعة أيضًا أقل بالنسبة للتكاليف الطبية المباشرة (15 دولار أمريكي) مقابل 34 دولارًا أمريكيًا، نسبة = 0.002) وغير طبية (30 دولارًا أمريكيًا) مقابل 54 دولارًا أمريكيًا، نسبة = 0.022) بين مرضى الكشف النشط عن الحالات. كانت تكاليف الانتشار الكارثي المباشر أقل بالنسبة لمرضى الكشف النشط عن الحالات لجميع العتبات. كما تم توثيق انخفاض شدة التكاليف الكارثية لمرضى الكشف النشط عن الحالات، على الرغم من أن الفرق لم يكن ذا دلالة إحصائية.

الاستنتاجات: يمكن أن يقلل الكشف النشط عن الحالات من التكاليف التي يتحملها المريض إلى حد كبير، مما يساهم في تحقيق الهدف من استراتيجية نهاية السل. يجب أيضًا تنفيذ سياسات تآزر أخرى، مثل الحماية الاجتماعية، لتقليل التكاليف الكارثية إلى الصفر بين الأسر المتضررة من السل.

Translated from English version into Arabic by Reem Jano, revised by Muhannad Al-Bayk, through

TRANSLATORS
WITHOUT BORDERS

病例主动发现在降低尼泊尔结核病患者灾难性支出中的作用

Suman Chandra Gurung, Kritika Dixit, Bhola Rai, Maxine Caws, Puskar Raj Paudel, Raghu Dhital, Shraddha Acharya, Gangaram Budhathoki, Deepak Malla, Jens W. Levy, Job van Rest, Knut Lönnroth, Kerri Viney, Andrew Ramsay, Tom Wingfield, Buddha Basnyat, Anil Thapa, Bertie Squire, Duolao Wang,

Gokul Mishra, Kashim Shah, Anil Shrestha, Noemia Teixeira de Siqueira-Filha

摘要

引言：世界卫生组织终止结核病策略设定的里程碑是在 2020 年没有因结核病造成灾难性支出的家庭。在全球范围内，病例主动发现（ACF）在降低患者支出方面的作用尚未确定。因此，本研究旨在比较结核患者主动发现（ACF）和被动发现（PCF）的成本，从而确定该病在尼泊尔患者承受的灾难性支出的发生率和强度。

方法：该研究于 2018 年 6 月至 8 月在尼泊尔 Bardiya 和 Pyuthan（第 5 省）开展。该研究纳入 100 例患者，其 PCF 和 ACF 的比例为 1∶1，即每个区各有 25 例 ACF 和 PCF 患者。采用 WHO 结核病患者费用核算工具（WHO TB patient costing tool）来收集患者或其家庭成员的间接/直接医疗和非医疗费用的信息。上述总费用超过患者家庭年总收入的 20%即为灾难性支出。本研究采用正过冲方法（positive overshoot method）计算灾难性支出的强度。卡方和 Wilcoxon-Mann-Whitney 检验用于比较比例和成本。同时，进行 Mantel Haenszel 检验来评估灾难性支出与病例发现方法之间的关联。

结果：共调查 99 例患者（50 例 ACF 和 49 例 PCF）。与 PCF 相比，通过 ACF 确诊的患者在治疗前的费用较低；ACF、PCF 的直接医疗费用分别为 14 美元和 32 美元（P = 0.001）；非直接医疗费用分别为 3 美元和 10 美元（P = 0.004）；间接的和时间损失分别为 4 美元和 13 美元（P < 0.001）。将治疗前和强化治疗阶段的支出合并计算，与 PCF 相比，ACF 患者的直接医疗（15 美元和 34 美元，P = 0.002）和非医疗（30 美元和 54 美元，P = 0.022）费用也较低。在所有阈值下，ACF 诊断的患者灾难性直接支出发生率均较低。除此之外，ACF 患者的灾难性支出的强度亦较低，但与 PCF 相比无统计学意义。

结论：主动病例发现可以大大降低患者的治疗费用，有助于终止结核病策略的目标。但是该目标的实现还需要其他政策，如社会保护等的协助。

Translated from English version into Chinese by Cong-Shan Liu, edited by Pin Yang

Le rôle du dépistage actif de cas dans la réduction des coûts considérables de la tuberculose pour le patient au Népal

Suman Chandra Gurung, Kritika Dixit, Bhola Rai, Maxine Caws, Puskar Raj Paudel, Raghu Dhital, Shraddha Acharya, Gangaram Budhathoki, Deepak Malla, Jens W. Levy, Job van Rest, Knut Lönnroth, Kerri Viney, Andrew Ramsay, Tom Wingfield, Buddha Basnyat, Anil Thapa, Bertie Squire, Duolao Wang, Gokul Mishra, Kashim Shah, Anil Shrestha, Noemia Teixeira de Siqueira-Filha

Résumé

Contexte: La stratégie WHO End TB de l'Organisation Mondiale de la Santé (OMS) pour éradiquer la tuberculose a posé un jalon pour réduire à zéro, d'ici 2020, le nombre de ménages touchés par la tuberculose (TB) et confrontés à des coûts beaucoup trop importants. Le rôle de la recherche active de cas (ACF) pour réduire les coûts des patients n'a pas été déterminé à l'échelle mondiale. Cette étude avait donc pour objectif de comparer les coûts supportés par les patients tuberculeux diagnostiqués par

recherche active de cas (ACF) la recherche passive de cas (PCF), et de déterminer la prévalence et l'intensité des coûts impressionnants pour les patients atteints de la tuberculose au Népal.
Méthodologie: Cette étude a été menée dans deux districts du Népal : Bardiya et Pyuthan (Province n° 5) entre juin et août 2018. Cent patients ont été retenus pour cette étude selon un ratio de 1:1 (PCF : ACF, 25 patients ACF consécutifs et 25 patients PCF consécutifs dans chaque district). L'outil de calcul OMS TB du coût des patients atteints de tuberculose a été utilisé pour collecter des informations concernant les coûts directs et indirects, médicaux et non médicaux, auprès des patients ou auprès d'un membre de leur famille. Les coûts élevés ont été calculés en fonction de la proportion de patients dont les frais totaux dépassent 20 % du revenu annuel de leur ménage. L'intensité des coûts onéreux a été calculée selon la méthode du dépassement positif. Les tests du Khi-deux et de Wilcoxon-Mann-Whitney ont été utilisés pour comparer les proportions et les coûts. En même temps, le test Cochran-Mantel-Haenszel a été effectué pour évaluer le lien entre les coûts exorbitants et le type de diagnostic.
Résultats: 99 patients ont été interrogés (50 ACF et 49 PCF). Les patients diagnostiqués à l'aide de l'ACF ont eu des coûts moindres pendant la période de pré-traitement (soins médicaux directs: 14 USD contre 32 USD, P = 0,001 ; soins directs non médicaux: 3 USD contre 10 USD, P = 0,004 ; indirect, temps perdu: 4 USD contre 13 USD, P < 0,001). Le coût des phases combinées de pré-traitement et intensive a également été plus modéré pour les coûts médicaux directs (15 USD vs 34 USD, P = 0,002) et non médicaux (30 USD vs 54 USD, P = 0,022) chez les patients ACF. La prévalence des coûts directs importants était plus faible chez les patients ACF, tous seuils confondus. Une intensité moindre des coûts élevés a également été observée chez les patients ACF, bien que la différence ne soit pas statistiquement significative.
Conclusions: Le dépistage actif des cas (ACF) peut réduire considérablement les coûts supportés par les patients, contribuant ainsi à atteindre l'objectif du projet End TB Strategy. D'autres politiques menées en synergie, telles que la protection sociale, devront également être mises en œuvre pour réduire à zéro les coûts inacceptables des ménages touchés par la tuberculose.

Translated from English version into French by Yasmine Zongo, revised by Clarisse Pean, through

TRANSLATORS
WITHOUT BORDERS

Роль ранего выявления заболевания туберкулезом в Непале в уменьшении катастрофических затрат, понесенных пациентами

Суман Чандра Гурунг, Критика Диксит, Бхола Рай, Максин Коуз, Пушкар Радж Паудель, Рагху Дхитал, Шраддха Ачарья, Гангарам Буддатоки, Дипак Малла, Дженс В. Леви, Джоб ван Рест, Кнут Лённрот, Керри Вайни, Эндрю Рамзай, Том Уингфилд, Будда Баснят, Анил Тхапа, Берти Сквайр, Дуолао Ван, Гокул Мишра, Кашим Шах, Анил Шреста, Ноемия Тейшейра де Сикейра-Фила

Аннотация

Общая информация: Стратегия Всемирной организации здравоохранения (ВОЗ) по борьбе с туберкулезом стала важной вехой в процессе снижения до нуля к 2020 году числа домохозяйств,

затронутых туберкулезом (ТБ) и несущих катастрофические расходы. Роль ранее выявление заболеваний (РВЗ) в снижении затрат, понесенных пациентами, еще не была определена в мировом масштабе. Поэтому это исследование было направлено на сравнение затрат, понесенных больными ТБ с ранним выявлением заболеваний (РВЗ) и больных с пассивным выявлением заболеваний (ПВЗ), и на определение распространенности и интенсивности катастрофических затрат, понесенных пациентами в Непале.

Методы: Исследование проводилось в двух районах Непала: Бардия и Пютан (Провинция № 5) в период с июня по август 2018 года. Сто пациентов были включены в это исследование в соотношении 1:1 (ПВЗ:РВЗ, последовательно 25 пациентов с РВЗ и последовательно 25 пациентов с ПВЗ в каждом районе). Инструмент ВОЗ для расчета затрат пациентов с ТБ был применен для сбора информации от пациентов или членов их семей касательно понесенных ими косвенных и прямых медицинских и немедицинских расходов. Катастрофические расходы были вычислены на основе доли пациентов, понесших общие расходы, превышающие 20% годового дохода их домохозяйства. Интенсивность катастрофических затрат была рассчитана с использованием метода положительного превышения (перепада). Проверки по критерию Хи-квадрат и Уилкоксона-Манна-Уитни использовались для сравнения пропорций и затрат. Между тем, был проведен тест Мантеля-Хэнзеля для оценки связи между катастрофическими затратами и типом диагноза.

Результаты: Были опрошеныевяносто девять пациентов (50 РВЗ и 49 ПВЗ). Пациенты с РВЗ понесли более низкие расходы в период до лечения (прямое медицинское обслуживание: 14 долларов США против 32 долларов США, P = 0,001; прямое немедицинское лечение: 3 доллара США против 10 долларов США, P = 0,004; непрямые затраты, потеря времени: 4 долл. США против 13 долл. США, P < 0,001). Стоимость комбинированных этапов предварительной терапии и интенсивной терапии также была ниже для прямой медицинской помощи (15 долл. США против 34 долл. США, P = 0,002) и немедицинских услуг (30 долл. США против 54 долл. США, P = 0,022) среди пациентов с РВЗ. Распространенность катастрофических прямых затрат была ниже среди пациентов с РВЗ для всех пороговых значений. Более низкая интенсивность катастрофических затрат была также задокументирована для пациентов с РВЗ, хотя разница не была статистически значимой.

Выводы: РВЗ может существенно сократить расходы, которые несут пациенты, что будет способствовать достижению цели Стратегии по ликвидации туберкулеза. Другие синергетические стратегии, такие как социальная защита, также должны быть реализованы для снижения катастрофических затрат до нуля среди домохозяйств, затронутых туберкулезом.

Translated from English version into Russian by Maria Petrenko, revised by Michael Orlov, through

TRANSLATORS
WITHOUT BORDERS

El papel de búsqueda activa de casos, en la reducción de los costos catastróficos que sufren los pacientes, por causa de la tuberculosis en Nepal

Suman Chandra Gurung,Kritika Dixit, Bhola Rai, Maxine Caws, Puskar Raj Paudel, Raghu Dhital, Shraddha Acharya, GangaramBudhathoki, Deepak Malla, Jens W. Levy, Job van Rest, Knut Lönnroth, Kerri Viney,Andrew Ramsay, Tom Wingfield, Buddha Basnyat, Anil Thapa,Bertie Squire,Duolao Wang, Gokul Mishra,Kashim Shah, Anil Shrestha, Noemia Teixeira de Siqueira-Filha

Resumen

Antecedentes: La Estrategia, para poner fin a la tuberculosis, por parte de la Organización Mundial de la Salud (OMS), ha establecido un hito, para reducir el número de hogares afectados por la tuberculosis (TB), de cara a los costos catastróficos, hasta 0 para el año 2020. El papel de la detección activa de casos (ACF; *por sus siglas en inglés*) en la reducción de gastos de los pacientes, no se ha determinado integralmente. Por lo tanto, el objetivo de este estudio fue comparar los gastos que sufren los pacientes con tuberculosis diagnosticados, por medio de la detección activa de casos y la detección pasiva de casos (PCF; *por sus siglas en inglés*), y así determinar la prevalencia e intensidad de los gastos catastróficos que sufren los pacientes, en Nepal.

Métodos: El estudio se llevó a cabo en dos distritos de Nepal: Bardiya y Pyuthan (Provincia nº5), entre junio y agosto de 2018. Se incluyeron a cien pacientes, en el presente estudio, en una proporción de 1:1 (PCF: ACF, 25 pacientes consecutivos de ACF y 25 pacientes consecutivos de PCF, en cada distrito). La herramienta de la OMS, para el cálculo de los gastos de los pacientes con tuberculosis se aplicó, para recopilar información de los pacientes o de un miembro de su familia sobre los gastos médicos y no médicos directos e indirectos. Los gastos catastróficos se calcularon en base a la proporción de pacientes, con gastos totales superiores al 20% de los ingresos anuales en sus respectivos hogares. La intensidad de los gastos catastróficos se calculó utilizando el método del rebasamiento positivo. Las pruebas chi-cuadrado y Wilcoxon-Mann-Whitney se utilizaron para comparar proporciones y costos. Mientras tanto, el Mantel Haenszeltest se realizó para evaluar la asociación entre los costos catastróficos y el tipo de diagnóstico..

Resultados: Se entrevistó a noventa y nueve pacientes (50 ACF y 49 PCF). Los pacientes diagnosticados, por medio de ACF, incurrieron en costos más bajos, durante el período de pre-tratamiento (médico directo: USD 14 vs USD 32, *P*-0.001; directo no médico: USD 3 vs USD 10, *P*-0.004; indirecto, pérdida de tiempo: USD 4 vs USD 13, $P < 0.001$). El costo de las fases de pre-tratamiento e intensivas combinadas también fue menor, para los costos médicos directos (USD 15 vs USD 34, *P*-0.002) y no médicos (USD 30 vs USD 54, *P*-0.022) entre los pacientes con ACF. La prevalencia de costos directos catastróficos fue menor para los pacientes con ACF, para todos los indicios. También se documentó una menor intensidad de los costos catastróficos, para los pacientes con ACF, aunque la diferencia no fue estadísticamente significativa.

Conclusiones: ACF puede reducir, sustancialmente, los costos incurridos por los pacientes, contribuyendo al objetivo de la Estrategia de Fin de la Tuberculosis. También será necesario aplicar otras políticas sinérgicas, como la protección social, para reducir los costos catastróficos a cero, entre los hogares afectados por la tuberculosis.

Translated from English version into Spanish by Paula Selgas, revised by Maria Luz Puerta, through

TRANSLATORS
WITHOUT BORDERS
