# Supplementary material for: The role of active case finding in reducing patient incurred catastrophic costs for tuberculosis in Nepal
Source: Infect Dis Poverty. 2019 Dec 3;8:99. doi: 10.1186/s40249-019-0603-z (PMC6889665; doi:10.1186/s40249-019-0603-z)
Supplement: Supplementary file 2 — Additional file 2. Algorithm for TB REACH active case finding through contact tracing, Nepal, 2018. [file 40249_2019_603_MOESM2_ESM.docx]

Additional file 2 Algorithm for TB REACH active case finding through contact tracing, Nepal, 2018.

Microscopic Centres (NTP)

Treatment Centres/ Sub Centres (NTP)

Index cases- Identification and Verification

Interview of Index cases**

Social contacts Contacts

Prioritization of Suspected Contacts

Screening of Suspected Contacts

Sputum Collection- Door to Door visit

Microscopic Examination- Microscopy Centres (NTP)

Sputum Smear Microscopy Negative (SS-)

Sputum Smear Microscopy Positive (SS+)

TB suspected

Enrollment into DOTS (NTP)

Follow up after 4 weeks

Follow up for Treatment Adherence

Basic Health Education on TB Prevention

Xpert MTB/RIF Testing

B- (Bacteriological Negative)

B+ (Bacteriological Positive), Rif Resistance

B+ (Bacteriological positive), Rif Sensitive
