# Supplementary material for: The role of active case finding in reducing patient incurred catastrophic costs for tuberculosis in Nepal
Source: Infect Dis Poverty. 2019 Dec 3;8:99. doi: 10.1186/s40249-019-0603-z (PMC6889665; doi:10.1186/s40249-019-0603-z)
Supplement: Supplementary file 3 — Additional file 3. Algorithm for TB REACH active case finding through TB camps and outpatient department of public hospitals, Nepal, 2018. [file 40249_2019_603_MOESM3_ESM.docx]

Additional file 3 Algorithm for TB REACH active case finding through TB camps and outpatient department of public hospitals, Nepal, 2018.

Follow up for treatment adherence

Screening in TB high risk areas through TB camps

Screening of presumptive TB patients in OPD visits

Microscopic Examination- Microscopy Centres (NTP)

Enrollment into DOTS (NTP)

TB suspected

B+ (Bacteriological Positive), Rif resistance

Basic health education on TB Prevention

Sputum smear microscopy positive (SS+)

Sputum smear microscopy negative (SS-)

B- (Bacteriological Negative)

Follow up after 4 weeks

B+ (Bacteriological positive), Rif sensitive

Identification and verification of patients through sputum smear microscopy/ Xpert MTB/RIF

Xpert MTB/RIF Testing
