# Supplementary figures and images for: The role of active case finding in reducing patient incurred catastrophic costs for tuberculosis in Nepal
Source: Infect Dis Poverty. 2019 Dec 3;8:99. doi: 10.1186/s40249-019-0603-z (PMC6889665; doi:10.1186/s40249-019-0603-z)

Additional file 4 TB REACH districts coverage and costing survey districts, Nepal, 2018.


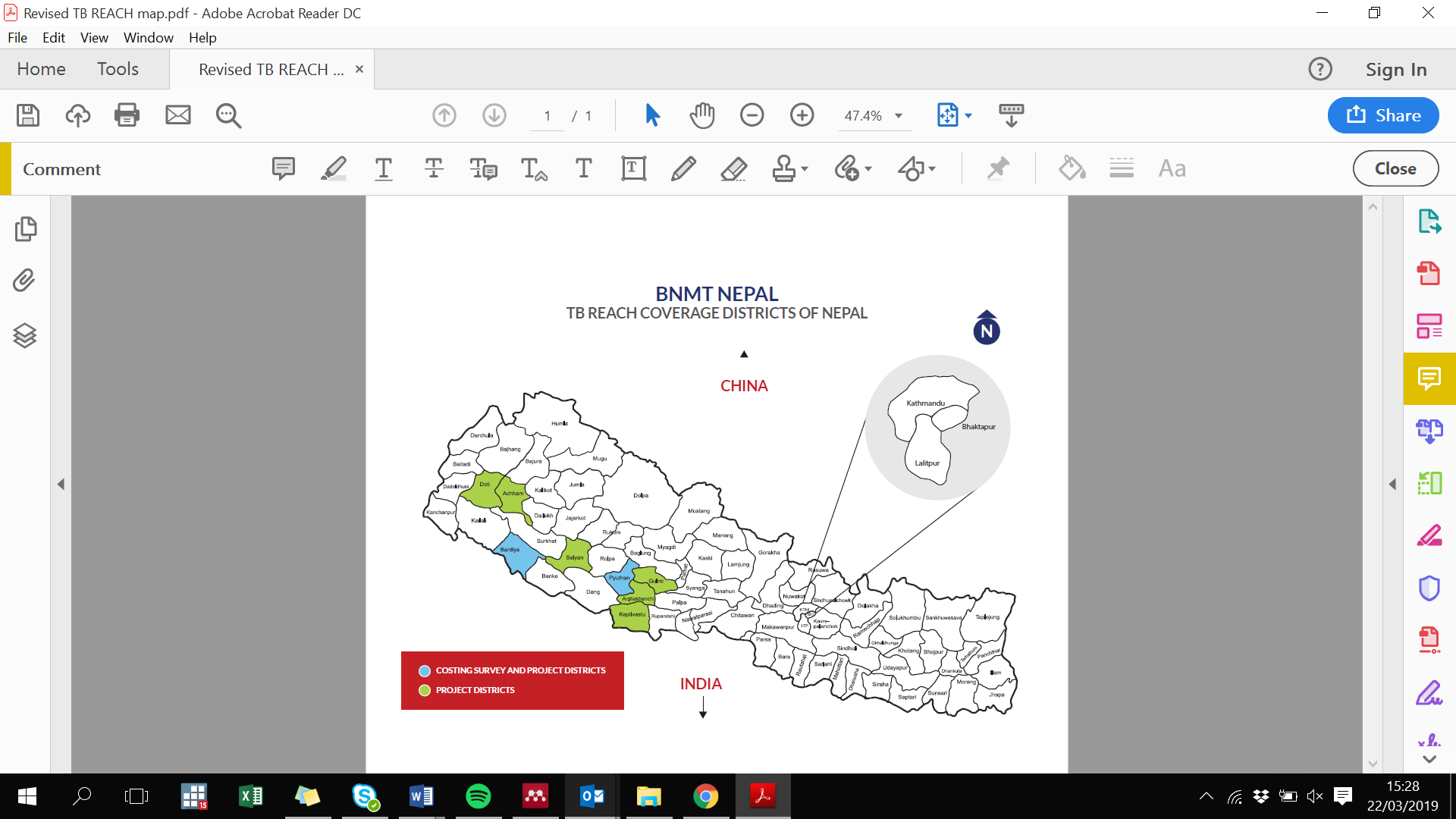

Supplement: Supplementary file 4 — Additional file 4. TB REACH districts coverage and costing survey districts, Nepal, 2018. [file 40249_2019_603_MOESM4_ESM.docx]
